# Development of the cephalopod-specific universal primer set and its application for the metabarcoding analysis of planktonic cephalopods in Korean waters

**DOI:** 10.7717/peerj.7140

**Published:** 2019-06-13

**Authors:** Eun-Bi Kim, Soo Rin Lee, Chung Il Lee, Hyun Park, Hyun-Woo Kim

**Affiliations:** 1 Interdisciplinary Program of Biomedical, Mechanical, and Electrical Engineering, Pukyong National University, Busan, Republic of Korea; 2 Department of Marine Bioscience, Gangneung-Wonju National University, Gangneung, Republic of Korea; 3 Unit of Polar Genomics, Korea Polar Research Institute, Incheon, Republic of Korea; 4 Department of Marine Biology, Pukyong National University, Busan, Republic of Korea

**Keywords:** Cephalopods, Metabarcoding, Next-generation sequencing, Paralarvae, Korean waters

## Abstract

Although spatiotemporal analysis of the cephalopod larvae provides the useful information for the effective management of their resources, it has been difficult mainly due to their low numbers in the mixed zooplankton net samples and difficulty in morphological identification. In order to analyze the planktonic cephalopods using next-generation sequencing (NGS), we have designed a cephalopod-specific universal (CPD) primer set targeting a region covering mitochondrial cytochrome b and ND6 genes based on the currently identified 36 complete cephalopod mitochondrial genome sequences in the GenBank database. The expected amplicon sizes by CPD primers were between 465 and 471 bp, which was applicable to the MiSeq system (Illumina, San Diego, CA, USA). NGS results of pooled DNAs from 8 months (including 739 zooplankton net samples) collected from Korean waters in 2016 showed the exclusive cephalopod sequences with little contaminant sequences supporting the specificity of CPD primer set. Total 47 representative cephalopod haplotypes (seven families and 10 genera) were obtained from 1,439,414 merged reads. Among the total analyzed haplotypes, *Watasenia scintillans*, *Todarodes pacificus*, and *Sepiola birostrata* were the most abundant species in Korean waters. Two “unidentified” clades in order Oegopsida were identified, which was showed less than 90% sequence identity but closely related to Enoploteuthidae and Idiosepiidae, respectively. Monthly changes in proportions of each haplotype were also identified, which may reflect its reproduction and spawning period. The larvae of *W. scintillans* was dominant from February to June, while high proportions of other cephalopod taxa were also identified from August to November. Only single haplotype was dominant in *W. scintillans* (Type 2) throughout the year, while two distinct haplotypes showed seasonal differences in *T. pacificus*.

## Introduction

Cephalopods are the third largest group of mollusks and occur throughout the ocean, ranging from the abyssal plain to the sea surface. Generally, their diversity decreases as the latitude increases (Nixon et al., 2003). Approximately 800 cephalopod species are currently known according to CephBase (http://cephbase.eol.org), many of which are commercially important. The management of commercially important cephalopod resources has been difficult, mainly owing to their short life span and sensitivity to environmental conditions (Emery, Hartmann & Gardner, 2016). For this reason, biological oceanographic studies have been undertaken in an attempt to understand the relationship between the distribution and abundance of cephalopod species and various environmental conditions, for example, oceanic current systems (Anderson & Rodhouse, 2001; Waluda et al., 2001). Correlations between the density of paralarvae (young cephalopods in the planktonic stages between hatchling and subadult) and adult catch biomass were shown for several species, suggesting that paralarval density indices could potentially be used to predict adult catch biomass (Kim & Lee, 2016; Rosa, Yamamoto & Sakurai, 2011; Sakurai et al., 2000).

Most previous studies of planktonic cephalopods have been based on morphological analyses of mixed zooplankton net samples (Bower & Takagi, 2004; Diekmann & Piatkowski, 2002; Moreno et al., 2008). These morphology-based approaches have several drawbacks. First, collecting planktonic cephalopods for quantitative analysis requires considerable time and effort because they have a low abundance in zooplankton net samples and patchy distributions (Boyle & Rodhouse, 2008; Vecchione et al., 2001). Second, the identification of most larval and juvenile stages of cephalopods is still confusing and problematic, and this has been the major obstacle in the study of cephalopod larval ecology. Furthermore, previous references for morphological identification of cephalopod paralarvae in Korean waters are very limited, making these studies even more difficult.

Morphological species identification is now being replaced by molecular-based approaches due to their fast and reliable results (Taberlet et al., 2012). One advantage of DNA-based identification is that researchers can analyze specific taxa from a variety of sources including zooplankton net samples, stomach contents, feces, or even environmental DNA samples (Berry et al., 2015; Chain et al., 2016; Hart et al., 2015; Jakubavičiūtė et al., 2017; Miya et al., 2015; Yoon et al., 2017). Advanced sequencing technologies such as next-generation sequencing (NGS) are accelerating a change in methodologies in marine ecological studies (Deiner et al., 2017; Lobo et al., 2017). The most widely used DNA markers for species identification have been either partial sequences of mitochondrial cytochrome c oxidase subunit I (COI) or ribosomal small subunits (SSU) (Armani et al., 2012; Folmer et al., 1994; Handy et al., 2011; Leray et al., 2013; Miya et al., 2015; Simon et al., 1994). However, application of general universal primers such as COI and SSU primers for the analysis of planktonic cephalopods is difficult due to high cross-amplification to other taxa present in the samples (Chapela et al., 2002; Giusti et al., 2017). Numbers of planktonic cephalopods are generally low and often undetected in NGS analysis by the outnumbered taxa in the zooplankton net samples, such as copepods or euphausiids. In order to obtain only cephalopod sequences from mixed samples, we developed a cephalopod-specific universal (CPD) primer set targeting the mitochondrial cytochrome b and ND6 regions that amplifies approximately 470 bp, the optimal size for the Illumina MiSeq system (Illumina, San Diego, CA, USA). The reliability of the CPD primer set was confirmed with both individual and zooplankton net samples and was finally applied to analyze the spatiotemporal biodiversity of planktonic cephalopods in Korean waters.

## Materials and Methods

### Cephalopod-specific universal primer set design

The CPD primer set was designed to satisfy three requirements. First, the CPD primers should avoid the cross-amplification of other zooplankton taxa in the mixed net samples such as copepods or euphausiids. Second, the amplicons should be shorter than the maximum read length of the Illumina MiSeq platform (2 × 300 bp). Third, the sequence variations of the CPD region should be high enough to distinguish each species. As result of multiple alignment with the 36 currently known complete cephalopod mitochondrial genome sequences (nine families) in the GenBank database, a pair of CPD primers (CPDfwd: 5′-GAYATYTGNCCYCADGG-3′ and CPDrvs: 5′-ATTTGYTAYTAYTGTGANGG-3′) were designed (Table 1). The CPD primers targeted the intergenic region between cytochrome b and ND6, a unique characteristic of cephalopod mitochondrial genome structure, generating PCR products of 465–471 bp in length, which is optimal for the Illumina MiSeq platform. In order to increase taxon coverage, five and four degenerate bases at the wobble position were included in CPDfwd and CPDrvs primers, respectively, and the highly conserved second codon was placed at the 3′ terminus of each primer (Table 1). All of the primers in this study were synthesized commercially (Macrogen, Seoul, Republic of Korea). In order to compare the sequence variation of the regions generated by three universal primer sets (CPD in present study, COI (Folmer et al., 1994), and 16S rRNA (Simon et al., 1994)), *p*-distances and Kimura 2-parameter distances were calculated using the MEGA7 program (Kumar, Stecher & Tamura, 2016).

**Table 1:** Multiple alignment of cephalopod-specific universal (CPD) primer set regions.

Family	CPDfwd	GenBank number	Sequence																				
			5′	-	G	A	Y	A	T	Y	T	G	N	C	C	Y	C	A	D	G	G	-	3′
Sepiidae	*Sepia pharaonis*	AP013076			•	•	T	•	•	C	•	•	A	•	•	C	•	•	A	•	•		
Sepiidae	*Sepia lycidas*	AP013075			•	•	T	•	•	T	•	•	A	•	•	T	•	•	A	•	•		
Sepiidae	*Sepia apama*	AP013073			•	•	T	•	•	T	•	•	A	•	•	C	•	•	A	•	•		
Sepiidae	*Sepia esculenta*	AB266516			•	•	T	•	•	T	•	•	A	•	•	T	•	•	A	•	•		
Sepiidae	*Sepia latimanus*	AP013074			•	•	T	•	•	T	•	•	A	•	•	T	•	•	A	•	•		
Sepiidae	*Sepia officinalis*	AB240155			•	•	T	•	•	C	•	•	A	•	•	T	•	•	T	•	•		
Sepiidae	*Sepiella inermis*	NC022693			•	•	T	•	•	T	•	•	A	•	•	T	•	•	A	•	•		
Sepiidae	*Sepiella maindroni*	KR912215			•	•	T	•	•	T	•	•	A	•	•	T	•	•	A	•	•		
Sepiidae	*Sepiella japonica*	AB675082			•	•	T	•	•	T	•	•	A	•	•	T	•	•	A	•	•		
Sepiolidae	*Semirossia patagonica*	AP013073			•	•	T	•	•	T	•	•	C	•	•	T	•	•	A	•	•		
Loliginidae	*Heterololigo bleekeri*	AB029616			•	•	T	•	•	T	•	•	C	•	•	T	•	•	A	•	•		
Loliginidae	*Uroteuthis edulis*	AB675081			•	•	T	•	•	T	•	•	A	•	•	T	•	•	A	•	•		
Loliginidae	*Uroteuthis duvaucelii*	KR051264			•	•	C	•	•	C	•	•	T	•	•	T	•	•	A	•	•		
Loliginidae	*Uroteuthis chinensis*	NC028189			•	•	T	•	•	T	•	•	A	•	•	C	•	•	A	•	•		
Loliginidae	*Loliolus uyii*	KP265013			•	•	C	•	•	T	•	•	A	•	•	T	•	•	A	•	•		
Loliginidae	*Loliolus beka*	NC028034			•	•	C	•	•	C	•	•	A	•	•	C	•	•	A	•	•		
Loliginidae	*Loliolus japonica*	NC030208			•	•	C	•	•	C	•	•	A	•	•	T	•	•	A	•	•		
Loliginidae	*Sepioteuthis lessoniana*	AB240154			•	•	T	•	•	T	•	•	A	•	•	T	•	•	A	•	•		
Loliginidae	*Doryteuthis opalescens*	KP336703			•	•	C	•	•	C	•	•	C	•	•	T	•	•	G	•	•		
Enoploteuthidae	*Watasenia scintillans*	AB240152			•	•	T	•	•	T	•	•	G	•	•	T	•	•	A	•	•		
Ommastrephidae	*Ommastrephes bartramii*	AB715401			•	•	C	•	•	T	•	•	A	•	•	C	•	•	A	•	•		
Ommastrephidae	*Todarodes pacificus*	AB240153			•	•	C	•	•	T	•	•	A	•	•	T	•	•	A	•	•		
Ommastrephidae	*Illex argentinus*	NC026908			•	•	T	•	•	T	•	•	A	•	•	T	•	•	A	•	•		
Ommastrephidae	*Dosidicus gigas*	EU068697			•	•	C	•	•	T	•	•	A	•	•	T	•	•	A	•	•		
Ommastrephidae	*Sthenoteuthis oualaniensis*	NC010636			•	•	T	•	•	T	•	•	C	•	•	C	•	•	A	•	•		
Bathyteuthidae	*Bathyteuthis abyssicola*	AP012225			•	•	C	•	•	C	•	•	A	•	•	T	•	•	A	•	•		
Idiosepiidae	*Idiosepius* sp.	KF647895			•	•	T	•	•	T	•	•	A	•	•	T	•	•	A	•	•		
Octopodidae	*Octopus minor*	HQ638215			•	•	T	•	•	T	•	•	A	•	•	T	•	•	T	•	•		
Octopodidae	*Octopus vulgaris*	AB158363			•	•	T	•	•	C	•	•	A	•	•	T	•	•	A	•	•		
Octopodidae	*Octopus conispadiceus*	NC029747			•	•	T	•	•	T	•	•	A	•	•	C	•	•	A	•	•		
Octopodidae	*Octopus bimaculatus*	NC028547			•	•	T	•	•	T	•	•	A	•	•	T	•	•	A	•	•		
Octopodidae	*Octopus bimaculoides*	KU295559			•	•	T	•	•	T	•	•	A	•	•	T	•	•	A	•	•		
Octopodidae	*Amphioctopus fangsiao*	AB240156			•	•	T	•	•	T	•	•	T	•	•	T	•	•	A	•	•		
Octopodidae	*Amphioctopus aegina*	NC029702			•	•	T	•	•	T	•	•	T	•	•	T	•	•	A	•	•		
Octopodidae	*Amphioctopus marginatus*	KY646153			•	•	T	•	•	T	•	•	A	•	•	C	•	•	A	•	•		
Vampyroteuthidae	*Vampyroteuthis infernalis*	AB266515			•	•	T	•	•	T	•	•	T	•	•	T	•	•	T	•	•		

Family	CPDrvs	GenBank number	Sequence																							
			5′	-	A	T	T	T	G	Y	T	A	Y	T	A	Y	T	G	T	G	A	N	G	G	-	3′
Sepiidae	*Sepia pharaonis*	AP013076			•	•	•	•	•	T	•	•	T	•	•	T	•	•	•	•	•	A	•	•		
Sepiidae	*Sepia lycidas*	AP013075			•	•	•	•	•	T	•	•	T	•	•	T	•	•	•	•	•	A	•	•		
Sepiidae	*Sepia apama*	AP013073			•	•	•	•	•	T	•	•	T	•	•	T	•	•	•	•	•	G	•	•		
Sepiidae	*Sepia esculenta*	AB266516			•	•	•	•	•	T	•	•	T	•	•	T	•	•	•	•	•	G	•	•		
Sepiidae	*Sepia latimanus*	AP013074			•	•	•	•	•	T	•	•	T	•	•	T	•	•	•	•	•	A	•	•		
Sepiidae	*Sepia officinalis*	AB240155			•	•	•	•	•	T	•	•	T	•	•	T	•	•	•	•	•	A	•	•		
Sepiidae	*Sepiella inermis*	NC022693			•	•	•	•	•	T	•	•	T	•	•	T	•	•	•	•	•	C	•	•		
Sepiidae	*Sepiella maindroni*	KR912215			•	•	•	•	•	T	•	•	T	•	•	T	•	•	•	•	•	G	•	•		
Sepiidae	*Sepiella japonica*	AB675082			•	•	•	•	•	T	•	•	T	•	•	T	•	•	•	•	•	A	•	•		
Sepiolidae	*Semirossia patagonica*	AP013073			•	•	•	•	•	T	•	•	T	•	•	T	•	•	•	•	•	A	•	•		
Loliginidae	*Heterololigo bleekeri*	AB029616			•	•	•	•	•	C	•	•	T	•	•	T	•	•	•	•	•	A	•	•		
Loliginidae	*Uroteuthis edulis*	AB675081			•	•	•	•	•	T	•	•	T	•	•	T	•	•	•	•	•	G	•	•		
Loliginidae	*Uroteuthis duvaucelii*	KR051264			•	•	•	•	•	T	•	•	T	•	•	T	•	•	•	•	•	G	•	•		
Loliginidae	*Uroteuthis chinensis*	NC028189			•	•	•	•	•	T	•	•	T	•	•	T	•	•	•	•	•	A	•	•		
Loliginidae	*Loliolus uyii*	KP265013			•	•	•	•	•	T	•	•	T	•	•	T	•	•	•	•	•	G	•	•		
Loliginidae	*Loliolus beka*	NC028034			•	•	•	•	•	T	•	•	T	•	•	T	•	•	•	•	•	G	•	•		
Loliginidae	*Loliolus japonica*	NC030208			•	•	•	•	•	T	•	•	T	•	•	T	•	•	•	•	•	G	•	•		
Loliginidae	*Sepioteuthis lessoniana*	AB240154			•	•	•	•	•	T	•	•	T	•	•	T	•	•	•	•	•	G	•	•		
Loliginidae	*Doryteuthis opalescens*	KP336703			•	•	•	•	•	C	•	•	T	•	•	C	•	•	•	•	•	G	•	•		
Enoploteuthidae	*Watasenia scintillans*	AB240152			•	•	•	•	•	T	•	•	T	•	•	T	•	•	•	•	•	G	•	•		
Ommastrephidae	*Ommastrephes bartramii*	AB715401			•	•	•	•	•	T	•	•	T	•	•	C	•	•	•	•	•	G	•	•		
Ommastrephidae	*Todarodes pacificus*	AB240153			•	•	•	•	•	T	•	•	T	•	•	C	•	•	•	•	•	G	•	•		
Ommastrephidae	*Illex argentinus*	NC026908			•	•	•	•	•	T	•	•	T	•	•	T	•	•	•	•	•	G	•	•		
Ommastrephidae	*Dosidicus gigas*	EU068697			•	•	•	•	•	T	•	•	T	•	•	T	•	•	•	•	•	G	•	•		
Ommastrephidae	*Sthenoteuthis oualaniensis*	NC010636			•	•	•	•	•	C	•	•	C	•	•	C	•	•	•	•	•	A	•	•		
Bathyteuthidae	*Bathyteuthis abyssicola*	AP012225			•	•	•	•	•	T	•	•	T	•	•	T	•	•	•	•	•	A	•	•		
Idiosepiidae	*Idiosepius* sp.	KF647895			•	•	•	•	•	T	•	•	T	•	•	T	•	•	•	•	•	A	•	•		
Octopodidae	*Octopus minor*	HQ638215			•	•	•	•	•	T	•	•	T	•	•	T	•	•	•	•	•	T	•	•		
Octopodidae	*Octopus vulgaris*	AB158363			•	•	•	•	•	T	•	•	T	•	•	T	•	•	•	•	•	G	•	•		
Octopodidae	*Octopus conispadiceus*	NC029747			•	•	•	•	•	T	•	•	T	•	•	T	•	•	•	•	•	A	•	•		
Octopodidae	*Octopus bimaculatus*	NC028547			•	•	•	•	•	T	•	•	T	•	•	T	•	•	•	•	•	A	•	•		
Octopodidae	*Octopus bimaculoides*	KU295559			•	•	•	•	•	T	•	•	T	•	•	T	•	•	•	•	•	G	•	•		
Octopodidae	*Amphioctopus fangsiao*	AB240156			•	•	•	•	•	T	•	•	T	•	•	T	•	•	•	•	•	G	•	•		
Octopodidae	*Amphioctopus aegina*	NC029702			•	•	•	•	•	T	•	•	T	•	•	T	•	•	•	•	•	G	•	•		
Octopodidae	*Amphioctopus marginatus*	KY646153			•	•	•	•	•	T	•	•	T	•	•	T	•	•	•	•	•	A	•	•		
Vampyroteuthidae	*Vampyroteuthis infernalis*	AB266515			•	•	•	•	•	T	•	•	T	•	•	C	•	•	•	•	•	G	•	•		

Note:

Each three bases represents a codon and degenerate nucleotide sites were shown by the IUPAC codes.

The taxon specificity and coverage of the CPD primers were tested by amplifying the genomic DNA of both individual cephalopod larvae and zooplankton net samples. The PCR mixture (20 μL) contained 200 ng template, 1 μL CPDfwd, and CPDrvs primers (50 pmol each), 2 μL dNTP (2.5 mM each), 2 μL 10× Ex Taq Buffer, 1 μL DMSO (99 %), 0.2 μL Ex Taq Hot Start Version (Takara Bio Inc., Kusatsu, Japan), and DNase/RNase-free deionized water. PCR cycles comprised denaturation at 94 °C for 3 min followed by 28 cycles of 94 °C for 30 s, 51 °C for 30 s, 72 °C for 30 s, and a final extension at 72 °C for 5 min. PCR products were stained with Loading STAR (Dyne Bio, Seongnam, Republic of Korea) and separated by electrophoresis in 1.5% agarose gel. Amplified products of the expected size were purified using the AccuPrep^®^ Gel Purification Kit (BIONEER, Daejeon, Republic of Korea). After gel purification, the DNA sequences of PCR products were commercially determined by Sanger sequencing (Macrogen, Seoul, Republic of Korea).

### Zooplankton sample collection and extraction of genomic DNA

**Figure 1:**
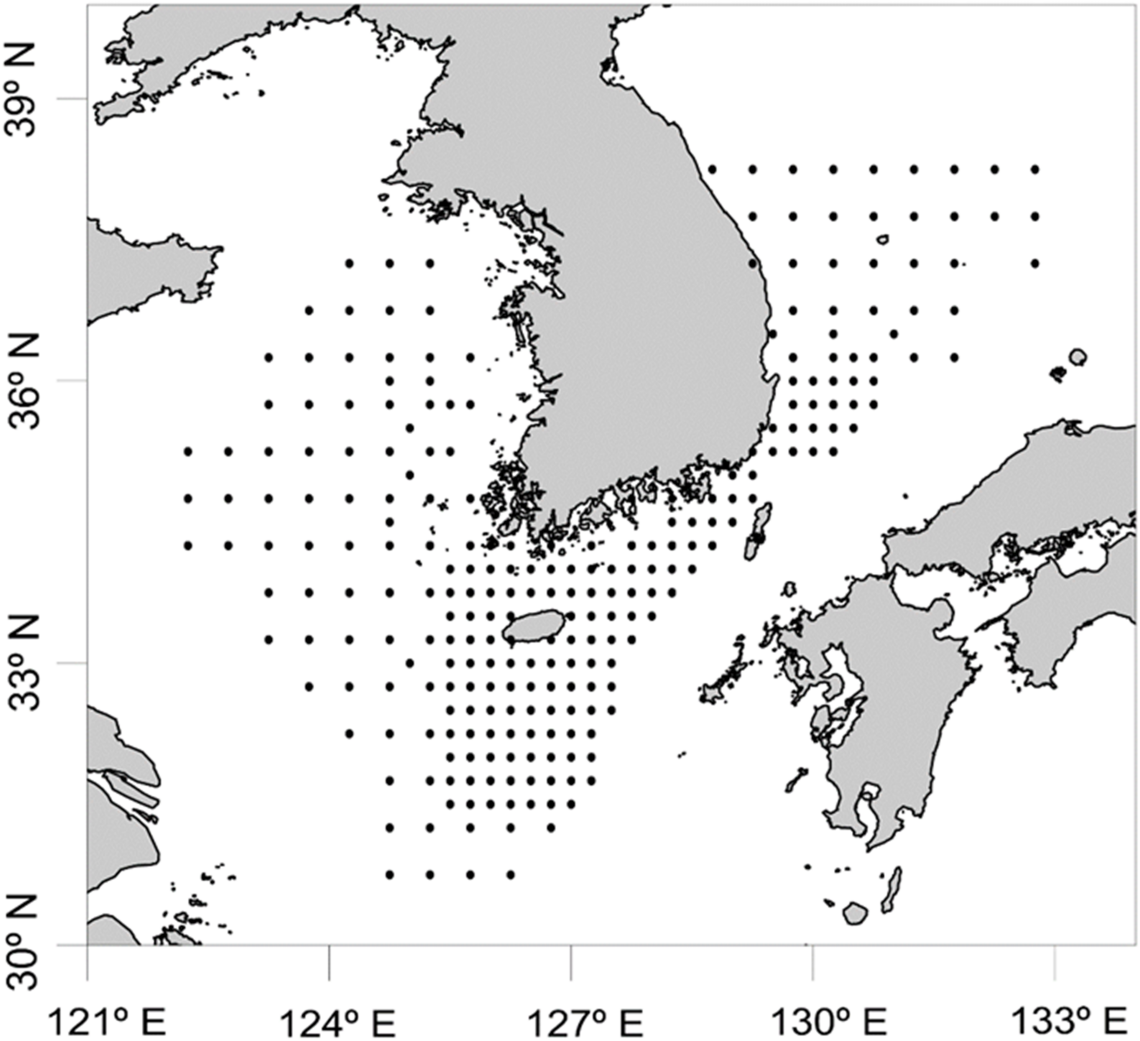
Sample collection sites for zooplankton in Korean waters, 2016.

In order to determine the biodiversity of planktonic cephalopods in Korean waters, 739 zooplankton net samples were collected over 8 months in 2016 as part of a regular plankton survey by the National Institute of Fisheries Science, and analyzed with the newly designed CPD primers (Fig. 1; Table S1). Sample collection was not possible during the months of January, July, September, and December owing to vessel maintenance and adverse weather. Each sample was collected by an oblique tow with a Bongo net (330 μm mesh size) and preserved in 99% ethanol (SK Chemicals, Seongnam, Republic of Korea). Prior to genomic DNA extraction, zooplankton samples were collected with a sieve (200 μm pore size), washed with tap water, dried briefly, and then transferred to conical tubes (50 mL in volume). After adding six volumes of Lysis Buffer (Biosesang, Seongnam, Republic of Korea) to the wet weight of each sample, the mixture was homogenized using a TissueLyser II (QIAGEN Korea, Seoul, Republic of Korea). Genomic DNA from each homogenized sample was extracted using the AccuPrep^®^ Genomic DNA Extraction Kit (BIONEER, Daejeon, Republic of Korea) according to the manufacturer’s instructions. The concentration of extracted genomic DNA was measured with a NanoDrop ND-1000 Spectrophotometer (Thermo Fisher Scientific, Waltham, MA, USA) and the extracted genomic DNA was aliquoted and stored at −80 °C until used for further experiments.

### Library preparation and sequencing using the MiSeq system

To determine the seasonal variation patterns of planktonic cephalopods, libraries for NGS analysis were constructed from the pooled PCR products generated from the samples for each month (total eight samples collected each month from February to November). A total of 739 genomic DNAs from different sample collection sites and times were used for NGS analysis. Libraries were constructed using the Nextera XT DNA Library Prep Kit (Illumina, San Diego, CA, USA). First, PCR amplifications were conducted with two adapter-linked CPD primers (NXTCPDfwd: 5′-TCGTCGGCAGCGTCAGATGTGTATAAGAGACAGGAYATYTGNCCYCADGG-3′ and NXTCPDrvs: 5′-GTCTCGTGGGCTCGGAGATGTGTATAAGAGACAGATTTGYTAYTAYTGTGANGG-3′), where adapter sequences are underlined. The PCR reaction mixture (20 μL) included 200 ng template, 1 μL of each NXTCPD primer (50 pmol), 2 μL dNTP (2.5 mM each), 2 μL of 10× Ex Taq Buffer, 1 μL DMSO (99%), 0.2 μL Ex Taq Hot Start Version (Takara Bio Inc., Kusatsu, Japan), and DNase/RNase-free deionized water. PCR cycles comprised denaturation at 94 °C for 3 min followed by 28 cycles of 94 °C for 30 s, 51 °C for 30 s, 72 °C for 30 s, and a final extension at 72 °C for 5 min. PCR products were stained with Loading STAR (Dyne Bio, Seongnam, Republic of Korea) and separated by electrophoresis in a 1.5% agarose gel. Amplified products with the expected size on the gel (approximately 535 bp) were cut and pooled together by month and purified using the AccuPrep^®^ Gel Purification Kit (BIONEER, Daejeon, Republic of Korea). Each purified product was indexed during a second PCR analysis using 1 μL of each index primer (10 pmol), 0.5 μL dNTP (10 mM each), 4 μL 5× Phusion HF Reaction Buffer (New England Biolabs, Hitchen, UK), 0.2 μL Phusion Hot Start Flex DNA polymerase (New England Biolabs, Hitchen, UK), and DNase/RNase-free deionized water in a 20 μL reaction volume. PCR cycles comprised 94 °C for 3 min followed by 15 cycles of 94 °C for 30 s, 55 °C for 30 s, 72 °C for 30 s, and a final extension of 3 min at 72 °C. Gel electrophoresis and gel purification were carried out as described above. The concentration of constructed libraries was measured by the Qubit dsDNA HS Assay Kit (Invitrogen, Carlsbad, CA, USA) and sequencing was carried out using the Illumina MiSeq platform (2 × 300 bp paired-end).

In order to compare the NGS results between CPD primers and metazoan universal COI primers, libraries were constructed with mlCOIintF and jgHCO2198 primer set (Leray et al., 2013). Three mlCOI libraries were prepared with 334 genomic DNAs in August, October, and November. The PCR conditions were as described in Aylagas et al. (2016) with slight modifications in template concentration and the number of PCR cycles. Other procedures for the library construction were identical to those used for the CPD primer set.

### Bioinformatic analysis

Raw MiSeq reads were trimmed to remove adapter/index sequences, and any regions with quality values under 20 and read lengths less than 100 nucleotides were discarded using CLC Genomics Workbench v. 8.0 (CLC Bio, Cambridge, MA, USA). The reads were merged with a minimum of seven bp overlap using Mother software v. 1.35.0 (Schloss et al., 2009). The merged reads were screened for the expected sizes and primer sequences were further trimmed to remove any mismatches. The removal of chimeric reads and clustering of merged reads were undertaken using UCHIME software v8.1 (Edgar et al., 2011). In order to obtain “representative haplotypes,” trimmed merged reads were then clustered with a cutoff sequence identity of 99.6%, and those with 10% or higher proportions were selected and added to the database. In order to identify cephalopod and metazoan haplotypes in Korean waters, trimmed merged reads were clustered with 98% cutoff sequence identity. Assignment of each haplotype was based on the sequence identity to hits in the NCBI-NT database using a local BLASTN search (http://www.ncbi.nlm.nih.gov). If the sequence identity of the query sequence and the top BLAST hit was more than or equal to 99%, the haplotype was assigned to the top-hit species. If the sequence identity was between 90% and 98%, the haplotype was assigned to the top-hit genus. Cephalopod and metazoan haplotypes with less than 90% identity were classified as “unidentified” and “unknown,” respectively. The assembled sequences assigned to cephalopod haplotypes with 98% identity were compared with representative haplotypes using local BLASTN. Haplotypes with less than two contigs were removed from further analysis. If the sequence identity was more than or equal to 98%, the haplotype was assigned to the representative haplotype name. Sequences that showed less than 98% identity with representative haplotypes were classified as “others.”

## Results

### Primer design and performance

**Figure 2:**
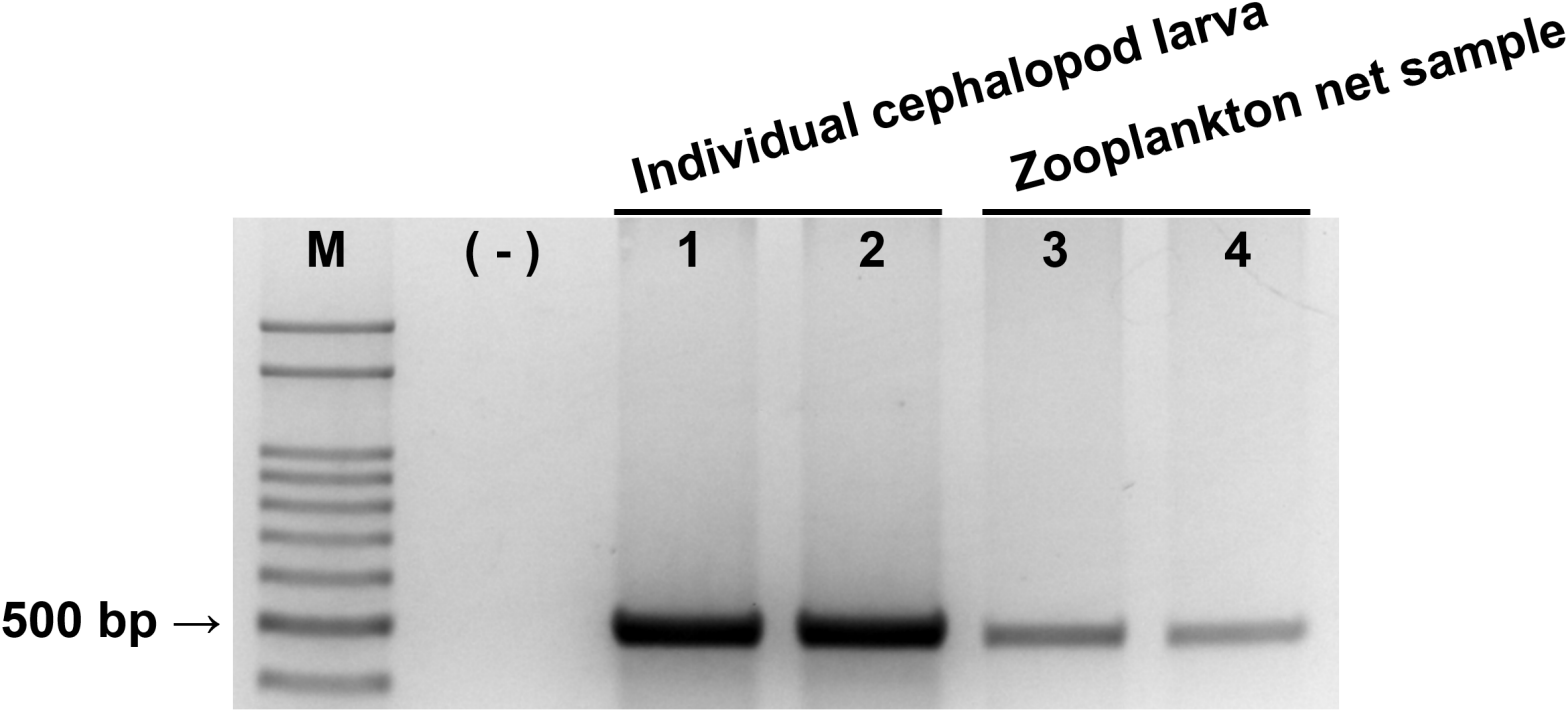
Evaluation of cephalopod-specific universal (CPD) primer set. Single PCR product amplified by CPD primers using two individual cephalopod larvae and two zooplankton net samples collected from Korean waters in August, 2016. M, 100 bp DNA ladder; (−), negative control; 1, *Todarodes pacificus*; 2, *Sepiola birostrata*; 3, Stn.72; 4, Stn.206.

The reliability of the newly designed CPD primer set was examined by PCR with individual cephalopod larvae and zooplankton net samples (Fig. 2). A single PCR product with the expected size (465–471 bp) was produced in both individual cephalopod larvae and zooplankton net samples. Sequences of all these PCR products also belonged to cephalopod species. These results support the reliability of the CPD primer set in terms of its specificity for cephalopod sequences from the mixed zooplankton samples.

Sequence variation of the region amplified by the CPD primers was compared with those amplified by the commonly used COI and 16S rRNA primers (Table 2). The mean *p*-distance and K2P distance of the CPD region at the genus level was 11.6% and 12.9%, respectively, which is between that of the 16S rRNA region (8.3% and 8.9%) and the COI region (13.7% and 15.4%). Similar results were also shown at family and order levels in both evolutionary models, indicating that the CPD region showed higher sequence variation than the 16S rRNA region but not as high as the COI region.

**Table 2:** Interspecific genetic diversities of three molecular markers in cephalopods based on *p*-distance and Kimura 2-parameter (K2P) model.

		CPD (present study)			COI Folmer et al. (1994)			16S rRNA Simon et al. (1994)		
		Mean (%)	Max. (%)	Min. (%)	Mean (%)	Max. (%)	Min. (%)	Mean (%)	Max. (%)	Min. (%)
*p*-distance	Order	13.1	20.4	0	15.5	21.6	0.2	8.4	13.8	0
	Family	12.7	19.4	0	15.2	19.6	0.2	8.1	13.8	0
	Genus	11.6	19.2	0	13.7	17.9	0.2	8.3	13.7	0
K2P distance	Order	14.6	24.2	0	17.5	26	0.2	8.9	15.3	0
	Family	14.1	22.5	0	17.2	22.9	0.2	8.7	15.3	0
	Genus	12.9	22.5	0	15.4	20.6	0.2	8.9	15.1	0

### MiSeq sequencing and taxonomic assignment

**Figure 3:**
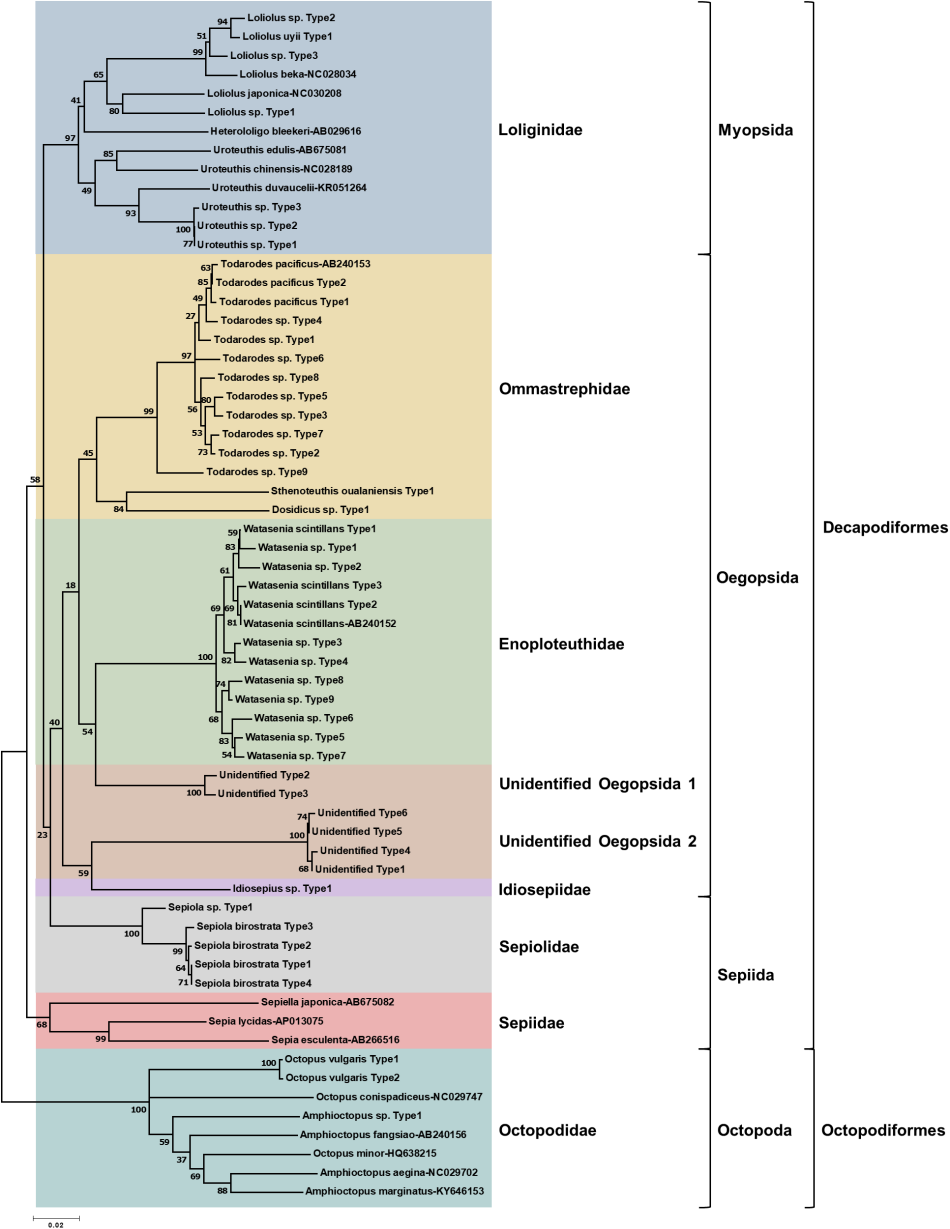
Phylogenetic analysis of representative cephalopod haplotypes in Korean waters, 2016. Phylogenetic tree was constructed by Minimum-evolution algorithm (MEGA version 7.0) under the 1,000 replication bootstrap.

To determine the monthly variation in the biodiversity of planktonic cephalopods in Korean waters, genomic DNA from 739 zooplankton net samples collected over a period of 8 months in 2016 was amplified by CPD primers and its amplicons were analyzed using the MiSeq sequencing system. After trimming the raw reads, a total of 1,439,414 merged reads were obtained from Korean waters, among which 47 representative haplotypes were determined at 99.6% cutoff sequence identity (Table S2). Their phylogenetic tree showed that CPD primers were successful in presenting both superorders, Decapodiformes, and Octopodiformes (Fig. 3). In the superorder Octopodiformes, three haplotypes (one in the genus *Amphioctopus* and two in the genus *Octopus*) were identified (Fig. 3). Two haplotypes showed 99% identity to *Octopus vulgaris* (GenBank number: AB158363), and the other showed only 90% identity to *Amphioctopus marginatus* (GenBank number: KY646153). In the superorder Decapodiformes, five families (Loliginidae, Ommastrephidae, Enoploteuthidae, Idiosepiidae, and Sepiolidae) and three orders (Myopsida, Oegopsida, and Sepiida) were identified in Korean waters (Fig. 3). Interestingly, two “unidentified” clades in the order Oegopsida were detected (Fig. 3). One clade (two haplotypes) was closely related to Enoploteuthidae, whereas the other (four haplotypes) was clustered with Idiosepiidae. Haplotypes in the unidentified clades showed the highest nucleotide sequence identity (88% and 84%) to *Bathyteuthis abyssicola* (GenBank number: AP012225) and *Illex argentinus* (GenBank number: KP336702), respectively. The two “unidentified” clades were named “unidentified Oegopsida 1” and “unidentified Oegopsida 2” (Fig. 3). Two genera in the family Loliginidae, *Loliolus* and *Uroteuthis*, were identified (Fig. 3). Among four haplotypes in the genus *Loliolus*, one showed 100% identity and two showed 98% identity to *Loliolus uyii* (GenBank number: KP265013). The fourth haplotype, *Loliolus* sp. Type 1, showed 92% sequence identity to *L. japonica* (GenBank number: NC030208). All three haplotypes in the genus *Uroteuthis* showed the highest identity (94%) to *Uroteuthis duvaucelii* (GenBank number: KR051264) (Fig. 3). Among the three clades in the family Ommastrephidae, the largest was *Todarodes pacificus* and its relatives, with 11 haplotypes (Fig. 3). The second clade exhibited 99% sequence identity to *Sthenoteuthis oualaniensis* (GenBank number: EU660577), and the third one showed the highest sequence identity (90%) to *Dosidicus gigas* (GenBank number: EU068697). A single genus in the family Enoploteuthidae, *Watasenia*, was identified in Korean waters, and all 12 haplotypes in the family showed highest sequence identity (97–100%) to *Watasenia scintillans* (GenBank number: AB240152) (Fig. 3). One haplotype showed the highest identity (95%) to *Idiosepius* sp. in the family Idiosepiidae (GenBank number: KF647895), but this species is not clearly established in taxonomic classification according to the World Register of Marine Species (WoRMS) (Fig. 3). In the family Sepiolidae, one genus clade represented *Sepiola birostrata* and its relative, *Sepiola* sp. (Fig. 3). We failed to identify any haplotype belonging to the family Sepiidae (Fig. 3). Overall, the CPD universal primer set successfully presented the biodiversity of cephalopod larvae in Korean waters, with a high degree of specificity for cephalopod species as well as a large taxon coverage.

### Monthly variation of planktonic cephalopods in Korean waters

**Figure 4:**
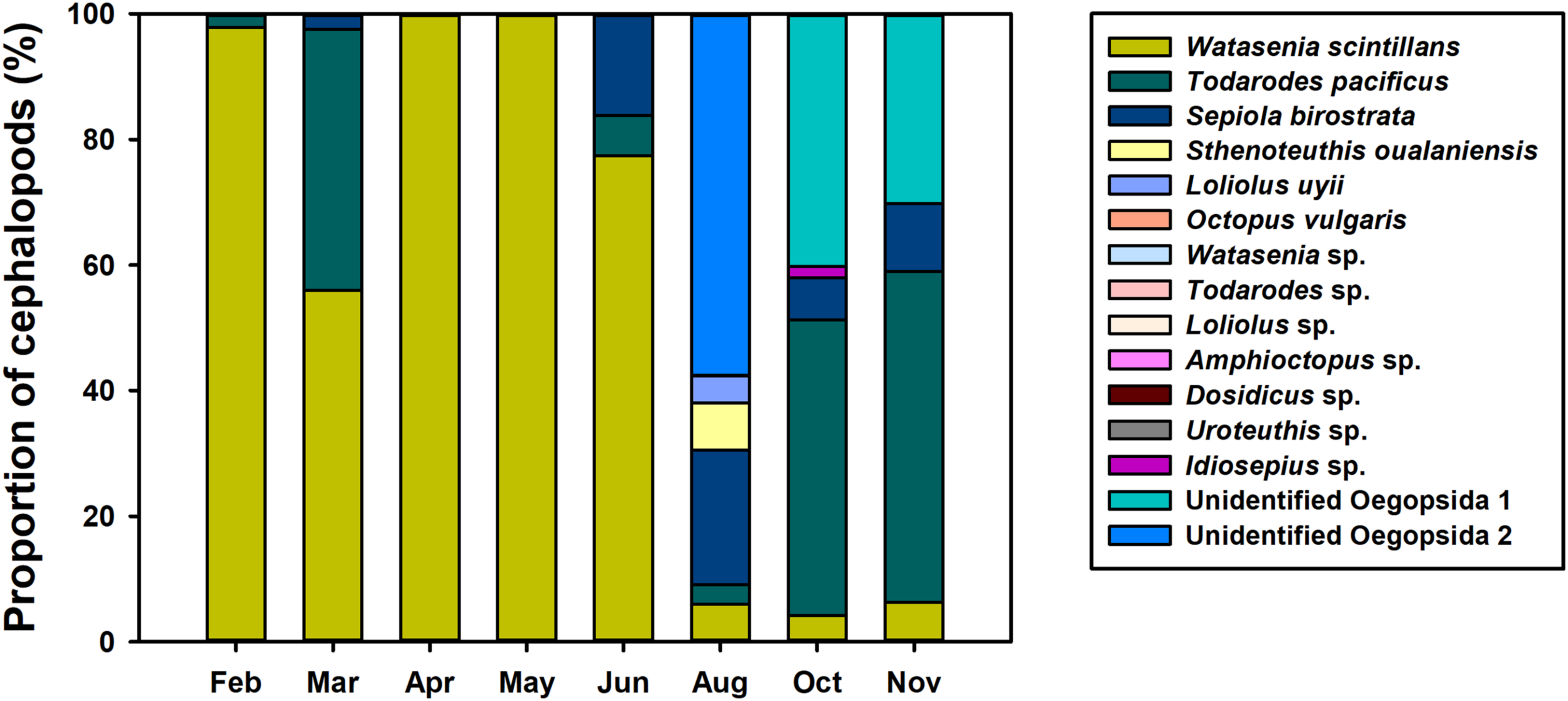
Proportions of planktonic cephalopod reads in Korean waters with different months as determined by NGS analysis.

To investigate the spatiotemporal distribution of planktonic cephalopods in Korean waters in 2016, monthly proportions of cephalopod haplotypes were analyzed (Fig. 4). A total of 1,440,241 contigs were obtained from the 739 zooplankton samples after clustering at 98% identity, and 48 haplotypes were identified by a 98% cutoff identity from the database that contains the representative haplotypes and sequences from the GenBank database (Table 3). Five haplotypes with low proportions (0.004%) showed less than 98% identity and were classified as “others” and excluded from further analysis (Table 3). The four most dominant planktonic cephalopods in this study were *W. scintillans, T. pacificus*, unidentified Oegopsida, and *Sepiola birostrata. W. scintillans* was dominant from February to June (from 55.92% to 99.97%) but occurred in lower proportions from August to November (from 4.20% to 6.24%) (Fig. 4; Table 3). The proportion of sequences identified as *T. pacificus* was especially high in March, October, and November (from 41.64% to 52.75%), whereas this species was not identified in April and May (Fig. 4; Table 3). Unidentified Oegopsida 1 was detected in high proportions in October and November (40.18% and 30.16%, respectively). Meanwhile, unidentified Oegopsida 2 was most dominant in August (57.52%) and its proportions were low in April, May, October, and November (from 0.007% to 0.03%) (Fig. 4; Table 3). The highest proportions of *Sepiola birostrata* were identified from June to November (from 6.65% to 21.49%) and were especially high in August (Fig. 4; Table 3). *Sthenoteuthis oualaniensis* was predominantly found in August (7.43%) and *L. uyii* was identified only in August, accounting for 4.36% (Fig. 4; Table 3). Despite low proportions, *O. vulgaris* was also detected in August (0.002%) and October (0.004%), and *Idiosepius* sp. was only detected in October, accounting for 1.78% (Fig. 4; Table 3).

**Table 3:** Summary of planktonic cephalopod haplotypes in Korean waters, 2016.

	February			March			April			May		
	Contigs	%	Haplotypes	Contigs	%	Haplotypes	Contigs	%	Haplotypes	Contigs	%	Haplotypes
*Watasenia scintillans*	107,830	97.845	1	61,514	55.924	1	85,853	99.977	1	206,811	99.954	1
*Todarodes pacificus*	2,286	2.074	1	45,805	41.643	1						
*Sepiola birostrata*	74	0.067	1	2,676	2.433	1	14	0.016	1			
*Sthenoteuthis oualaniensis*												
*Loliolus uyii*												
*Octopus vulgaris*												
*Watasenia* sp.												
*Todarodes* sp.										3	0.001	1
*Loliolus* sp.												
*Amphioctopus* sp.												
*Dosidicus* sp.	15	0.014	1									
*Uroteuthis* sp.												
*Idiosepius* sp.												
Unidentified Oegopsida 1										50	0.024	1
Unidentified Oegopsida 2							6	0.007	1	43	0.021	1
Others												
Total	110,205	100	4	109,995	100	3	85,873	100	3	206,907	100	4

	June			August			October			November		
	Contigs	%	Haplotypes	Contigs	%	Haplotypes	Contigs	%	Haplotypes	Contigs	%	Haplotypes
*Watasenia scintillans*	410,734	77.427	1	10,252	6.002	1	3,276	4.209	1	9,244	6.241	1
*Todarodes pacificus*	33,709	6.354	1	5,236	3.065	1	36,640	47.073	1	78,145	52.755	1
*Sepiola birostrata*	86,018	16.215	1	36,711	21.491	2	5,178	6.652	2	15,923	10.749	1
*Sthenoteuthis oualaniensis*				12,702	7.436	2	16	0.021	1			
*Loliolus uyii*				7,451	4.362	1						
*Octopus vulgaris*				3	0.002	1	3	0.004	1			
*Watasenia* sp.										42	0.028	1
*Todarodes* sp.	8	0.002	1									
*Loliolus* sp.				26	0.015	2						
*Amphioctopus* sp.				169	0.099	1						
*Dosidicus* sp.												
*Uroteuthis* sp.							47	0.060	1	8	0.005	1
*Idiosepius* sp.							1,386	1.781	1			
Unidentified Oegopsida 1							31,280	40.187	1	44,678	30.162	1
Unidentified Oegopsida 2				98,258	57.522	2	11	0.014	1	45	0.030	1
Others	7	0.001	1	11	0.006	2				44	0.030	2
Total	530,476	100	5	170,819	100	15	77,837	100	10	148,129	100	9

**Figure 5:**
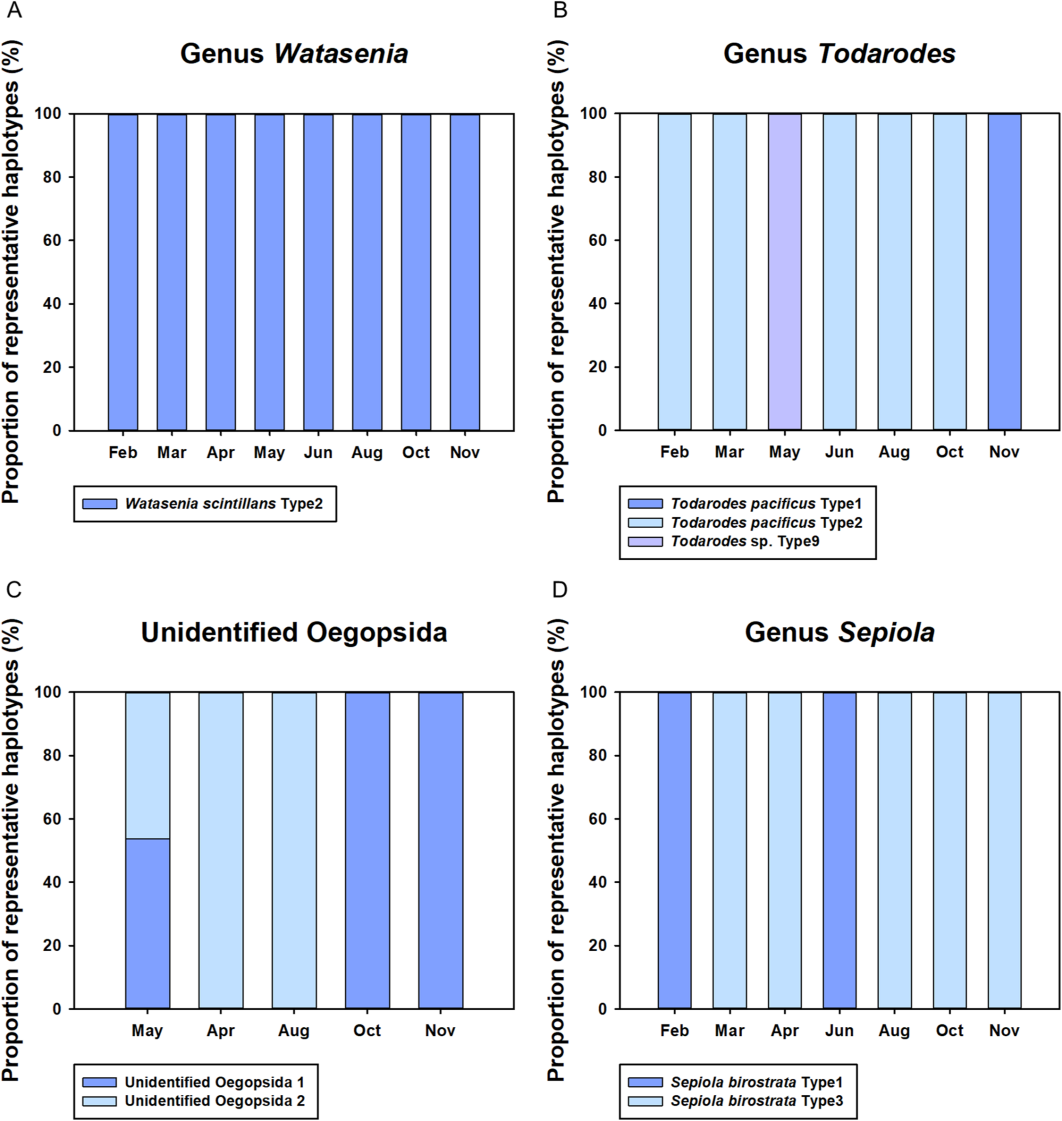
Proportions of representative haplotypes recovered from four dominant cephalopod genera in Korean waters. (A) Genus *Watasenia*; (B) genus *Todarodes*; (C) unidentified Oegopsida; (D) genus *Sepiola*.

Monthly changes in the proportions of representative haplotypes were analyzed for the dominant planktonic cephalopods in Korean waters (Fig. 5). In the genus *Watasenia*, *W. scintillans* Type 2 exhibited dominant proportions throughout our study period (Fig. 5). In contrast, we were able to see clear seasonal differences in the genus *Todarodes* haplotypes (Fig. 5). Among three representative haplotypes, *T. pacificus* Type 2, which showed 99% sequence identity to *T. pacificus* (GenBank number: AB240153), was dominant in February, March, June, August, and October. Two other haplotypes, *T. pacificus* Type 1 and *Todarodes* sp. Type 9, were dominant in November and May, respectively. Unidentified Oegopsida 1 was dominant in October and November (Fig. 5), whereas unidentified Oegopsida 2 was dominant in April and August. Both Oegopsida haplotypes occurred in May, accounting for 53.76% and 46.24%, respectively. In the genus *Sepiola*, *Sepiola birostrata* Type 3 was dominant in March, April, August, October, and November, whereas Type 1 was dominant in February and June (Fig. 5).

**Figure 6:**
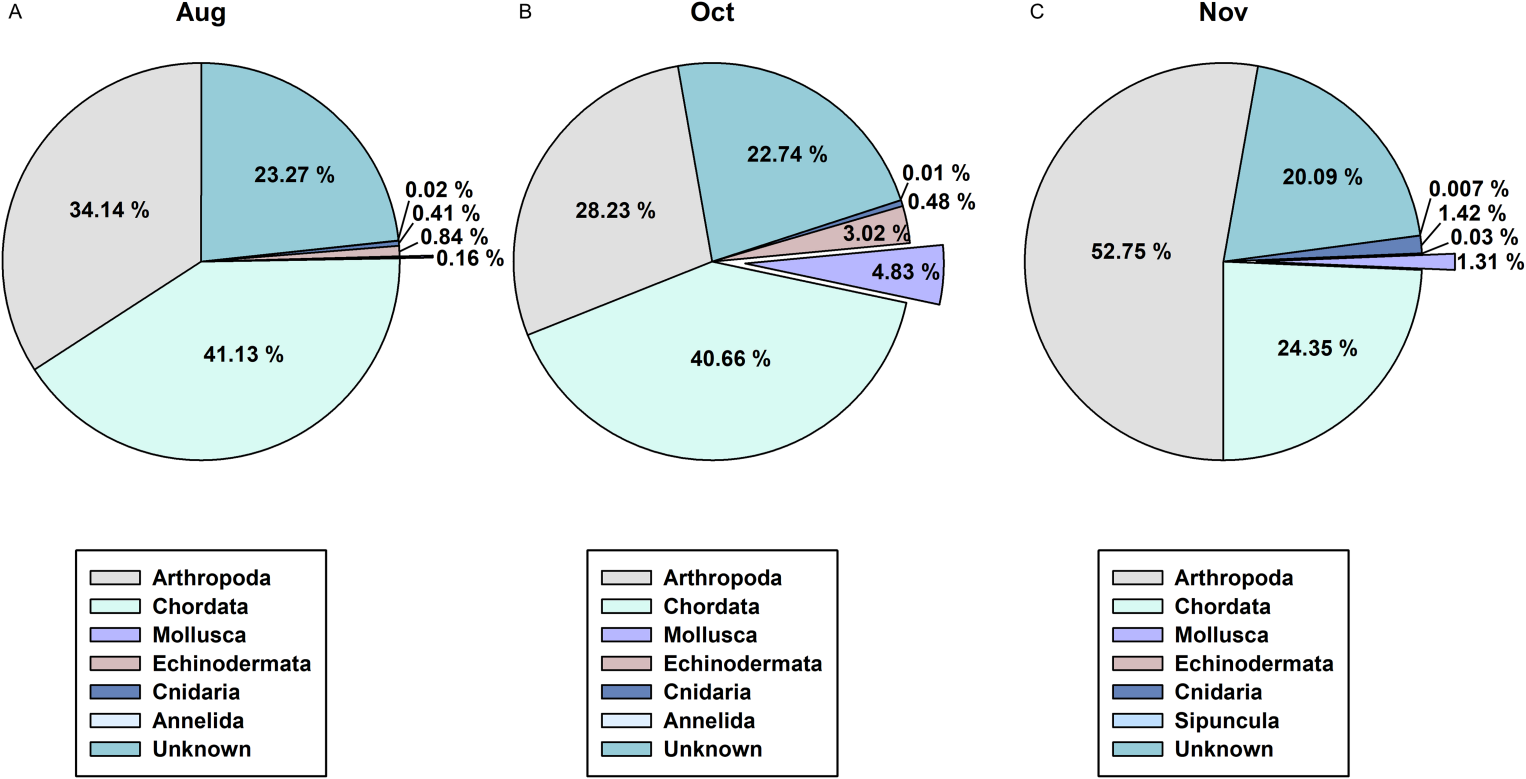
Proportions of metazoan phyla in the zooplankton net samples by the metabarcoding analysis with COI universal primer set. (A) August; (B) October; (C) November.

In order to determine the difference between the developed CPD primers and the typically used COI metabarcoding primers in presenting cephalopod sequences from the zooplankton net samples, we compared the NGS read data for the 3 months of August, October, and November (Fig. 6). Among the 1,142 metazoan haplotypes identified by mlCOIintF and jgHCO2198 primers (Leray et al., 2013) at 98% cutoff identity, molluscan reads accounted for 0.16%, 4.83%, and 1.31% in August, October, and November, respectively (Fig. 6). Among them, cephalopod species comprised only 0.01% (*Sthenoteuthis oualaniensis*, Octopodidae), 0.05% (*T. pacificus*), and 0.07% (*T. pacificus*, *Abralia andamanica*) of the total contigs in each analyzed month. Compared with the numbers of haplotypes obtained with the CPD primer set (15 in August, 10 in October, and 9 in November), only one in August (*Sthenoteuthis oualaniensis*) and in October (*T. pacificus*), and two in November (*T. pacificus*, *Abralia andamanica*) were identified by COI primer set. These results indicate that the newly designed CPD primer set is highly reliable for analyzing the biodiversity of cephalopods with high specificity.

## Discussion

### Reliability of the cephalopod-specific universal primer set

In the present study, we designed a CPD primer set to identify cephalopod species from mixed samples such as zooplankton net samples. The CPD primer set successfully amplified a single PCR product from both individual cephalopod larvae and mixed zooplankton net samples, and the NGS results showed only cephalopod sequences, indicating no cross-amplification from other taxa (Fig. 2; Table 3). The obtained NGS sequences also exhibited a wide range of cephalopod taxon coverage including both superorders, Decapodiformes and Octopodiformes, which also covers seven families (Fig. 3). We also showed that the commonly used COI universal primer set (Leray et al., 2013) failed to present cephalopod larval sequences as precisely as the CPD primer set (Fig. 6). These results suggest that the newly designed CPD primers will be a useful tool to identify planktonic cephalopods from zooplankton net samples without the need for manual selection or morphological observation, which are time-consuming and labor-intensive. A metabarcoding strategy with the CPD primers would enable researchers to design large-scale and long-term cephalopod surveys with relatively low cost and effort. However, the current metabarcoding strategy does not provide physical measurements such as larval size or developmental stage. In the present study, the data obtained were most likely from mesoplanktonic cephalopods because we excluded macroplanktonic cephalopods (>2 cm) by sieving and microplanktonic cephalopods by the plankton net mesh size (330 μm). Other researchers should ensure that their experimental design for metabarcoding and morphological analyses is suitable for their intended purpose.

Although the CPD primers exhibited a high degree of specificity and wide coverage for cephalopod taxa, several weak points were also identified in this study. First, the nucleotide sequence data amplified by the CPD primer set is limited compared with that of the currently used COI universal primers. Only 36 CPD sequences were available, as compared with more than 110 cephalopod COI sequences. In this study, 87.77% of the merged reads were identified at the species level with a cutoff of 99% sequence identity, while 12.10% of sequences from Korean waters samples were still classified as “unidentified” in 2016. The supplementation of CPD sequence data would facilitate further species-level identification and would require only a small-time investment, considering the low number of cephalopod species. Second, interspecific sequence diversity in the CPD region was not as high as in the COI region (Table 2). For example, *Sepiella japonica* (GenBank number: AB675082) and *Sepiella maindroni* (GenBank number: KR912215) were indistinguishable by CPD and 16S rRNA sequences but could be distinguished by the COI region. It may be possible to identify a greater number of species using the COI primer set; however, the high taxon specificity of the CPD primers allows for the detection of extremely low-numbered planktonic cephalopods, which cannot be achieved using the COI universal primer set. In addition, the CPD primers showed no cross-amplification of other plankton taxa, meaning that they can not only be used for qualitative analyses but also for quantitative analyses such as qPCR or proportional NGS analysis. With supplementation of the CPD sequence database, the CPD primer set will provide a good alternative method for the molecular metabarcoding analysis of cephalopod species.

### Identification of cephalopod species in Korean waters by NGS analysis

The planktonic cephalopods identified from 739 zooplankton net samples in Korean waters included haplotypes of seven families and 10 genera (Fig. 3). A total of 47 representative haplotypes, corresponding to at least 13 species, were identified in this study (Fig. 3). The putative number of cephalopod species identified seems low considering the large sample number and the fact that approximately 38 cephalopod species are currently reported to occur in Korean waters (Kim & Chun, 2010). There are several possible explanations for the low number of species identified in this study. First, the sample collection sites were mostly offshore; therefore, we predominantly detected species that drift in the currents around the Korean peninsula, such as *W. scintillans* and *T. pacificus* (Table 3; Fig. 4). The low number of species can also be attributed to the ecological characteristics of individual cephalopod species, some of which may be difficult to detect by Bongo net sampling. For example, we did not detect any species from the family Sepiidae, which are benthic or benthopelagic (Boletzky, 1983), even though some of these species are commercially important. Unlike the Octopodidae, which enter the plankton stage immediately after hatching, Sepiidae species stay on the bottom without coming up to the surface after hatching (Nixon & Mangold, 1998). Another possible explanation for the detection of low species numbers is that the CPD primer set might have low taxon coverage. This seems unlikely, however, as the taxonomic diversity detected by the CPD primers covered both cephalopod superorders, Decapodiformes, and Octopodiformes (Fig. 3). Further studies that include samples from coastal waters may help to explain the low number of species identified in the current study.

We have used the terms “haplotype” and “representative haplotype” instead of “operational taxonomic unit (OTU).” The term “OTU” was originally developed in the context of numerical taxonomy for situations where a robust taxonomic hypothesis is difficult to consult such as for meiofaunal specimens (Blaxter et al., 2005; Sokal & Sneath, 1963). However, considering the small number of cephalopod species (∼800 species worldwide), we believe that it is more useful to upload dominant haplotypes into the database rather than the artificial classification by sequence identity. It is likely that further studies will allow for a clearer identification of species, and “representative haplotypes” will provide more useful information regarding planktonic cephalopods in Korean waters than artificially clustered OTUs.

For some of the representative cephalopod haplotypes, we were able to estimate the species. For example, the *Todarodes* sp. Haplotypes, from the family Ommastrephidae, were likely to be *T. pacificus* as this is the only reported species in the genus *Todarodes* in Korean waters (Kim & Chun, 2010). However, *Todarodes* sp. Type 9 showed 95% sequence identity to *T. pacificus*, suggesting that it is likely to be a different species. According to WoRMS, the only species in the genus *Dosidicus* is *D. gigas*, which is endemic to the eastern Pacific (Nigmatullin, Nesis & Arkhipkin, 2001). Therefore, the *Dosidicus* sp. haplotype in Korean waters might belong to another genus within the family Ommastrephidae. In the family Enoploteuthidae, all of the sequences identified as *W. scintillans* and relatives are likely to be *W. scintillans*, as this is the only known species in the genus. In the family Idiosepiidae, different phylogenetic relationships were found depending on the gene sequences used (Takumiya et al., 2005). They appeared to be closely related to the order Oegopsida based on mitochondrial COIII gene sequences (Bonnaud, Boucher-Rodoni & Monnerot, 1997) or the order Myopsida (family Loliginidae) based on mitochondrial COI, 12S rRNA, 16S rRNA, and nuclear 28S rRNA gene sequences (Nishiguchi, Lopez & Boletzky, 2004). *Sepiola* was the only genus identified in the family Sepiolidae, and *Sepiola* sp. Type 1 formed a separate clade, suggesting that it may be a different species from the other *Sepiola* sequences detected.

Although the CPD region showed lower sequence variation than the COI region (Folmer et al., 1994), we were able to obtain some information about the genetic diversity of the major planktonic cephalopods in Korean waters. In the genus *Watasenia*, *W. scintillans* Type 2 was dominant throughout our study period, suggesting a single major haplotype (Fig. 5). Meanwhile, three different haplotypes (*T. pacificus* Type 1, Type 2, and *Todarodes* sp. Type 9) of the genus *Todarodes* were most abundant in our samples of 2016 (Fig. 5). Further studies are needed to assess which haplotypes occur repeatedly in Korean waters each year. Furthermore, although three cohorts of *T. pacificus* are currently reported in Korean waters (summer, autumn, and winter) and each cohort has independent migration patterns and distribution of spawning grounds (Kang et al., 1996; Kim & Kang, 1995; Sakurai et al., 2000), further studies are needed to investigate the relationship between the three haplotypes and the cohorts. Two unidentified Oegopsida were detected in May 2016, and these two putative species accounted for approximately half of the total sequences detected (Fig. 5). Different haplotypes within each unidentified Oegopsida may have originated from different species or populations. Each of the two *Sepiola birostrata* haplotypes occurred in different proportions in our study period, suggesting that they might be derived from different populations (Fig. 5). A regional long-term survey of these cephalopod haplotypes should be undertaken.

Monthly changes in the proportions of cephalopod haplotypes appears to reflect their spawning seasons in Korean waters (Fig. 4). Although *W. scintillans* was identified throughout the year, its paralarvae were most abundant from February to June and decreased significantly from August (Fig. 4). This pattern was similar to that found in a previous study that indicated a year-round spawning period, with the main spawning event taking place from April to July (Kawano, 2007). The highest proportion of *T. pacificus* was detected in November, followed by October and March, which is consistent with the fact that the autumn and winter cohorts accounted for most of their biomass (Kawabata et al., 2006). Although it is not possible to determine the relationship between genetic variation and cohorts of *T. pacificus* in Korea from the available data, it is worth noting that the proportion of *T. pacificus* Type 1 was highest only in November, whereas *T. pacificus* Type 2 was dominant in all other months examined. Based on results from the current and previous studies, we propose that *T. pacificus* Type 2 detected from June to October may be the summer cohort, whereas Type 1, detected in November, appears to be the autumn cohort (Fig. 5). *T. pacificus* Type 2, detected in high proportions from February to March, may be the winter cohort. However, there was no sequence difference in the CPD region between the *T. pacificus* Type 2 detected from June to October and those detected from February to March. Therefore, further study is needed to determine whether there is any genetic difference between the summer and winter *T. pacificus* Type 2 cohorts (Fig. 5). Although *Todarodes* sp. Type 9 was most abundant in May, it only accounted for a negligible proportion of the total cephalopod sequences, suggesting that this haplotype may not be the major squid in Korean waters and may stem from small numbers of paralarvae drifting along with the Tsushima Current, a branch of the Kuroshio Current. *Sthenoteuthis oualaniensis* was detected only in August and October in this study; however, based on a maturation study, this may spawn all year round with the peak spawning season occurring between March and May (Xinjun et al., 2007) (Fig. 4). Further study is needed to determine the relationship between the spatiotemporal distribution and abundance and the reproduction and catches of cephalopods.

## Conclusion

We have developed a CPD primer set and analyzed cephalopod diversity in zooplankton samples collected from Korean waters in 2016. The CPD primer set exclusively amplified cephalopod sequences, supporting its specificity to cephalopods. From NGS results, we were able to obtain 48 cephalopod haplotypes belonging to seven families and 10 genera. These results confirm that metabarcoding analysis using the CPD primer set is a good alternative strategy for planktonic cephalopod surveys, presenting both qualitative and quantitative data. We were also able to estimate the genetic diversity of cephalopod species using the CPD primers. As further data accumulate, we will be able to obtain useful information related to many ecological events such as spawning, migration, and recruitment, which will be useful for the scientific management of cephalopod resources in Korean waters.

## Supplemental Information

10.7717/peerj.7140/supp-1 Supplemental Information 1 Sample stations in this study.

10.7717/peerj.7140/supp-2 Supplemental Information 2 Summary of 47 representative cephalopod haplotypes identified in Korean waters.

10.7717/peerj.7140/supp-3 Supplemental Information 3 Evaluation of cephalopod-specific universal (CPD) primer set. Single PCR product amplified by CPD primers using two individual cephalopod larvae and two zooplankton net samples collected from Korean waters in August, 2016. M, 100 bp DNA ladder; (-), negative control; 1, *Todarodes pacificus*; 2, *Sepiola birostrata*; 3, Stn.72; 4, Stn.206.

## Additional Information and Declarations

### Field Study Permissions

The following information was supplied relating to field study approvals (i.e., approving body and any reference numbers): Field experiments were approved by the National Institute of Fisheries Science.

### DNA Deposition

The following information was supplied regarding the deposition of DNA sequences: The newly identified two haplotype sequences in this study are accessible via GenBank accession numbers MH165176 and MH165177.

### Data Availability

The following information was supplied regarding data availability: Kim, Eun-Bi (2019): Development of the cephalopod-specific universal primer set and its application for the metabarcoding analysis of planktonic cephalopods in Korean waters. figshare. Dataset. https://doi.org/10.6084/m9.figshare.7856708.v1
