# Supplementary figures and images for: Development of the cephalopod-specific universal primer set and its application for the metabarcoding analysis of planktonic cephalopods in Korean waters
Source: PeerJ. 2019 Jun 13;7:e7140. doi: 10.7717/peerj.7140 (PMC6571131; doi:10.7717/peerj.7140)

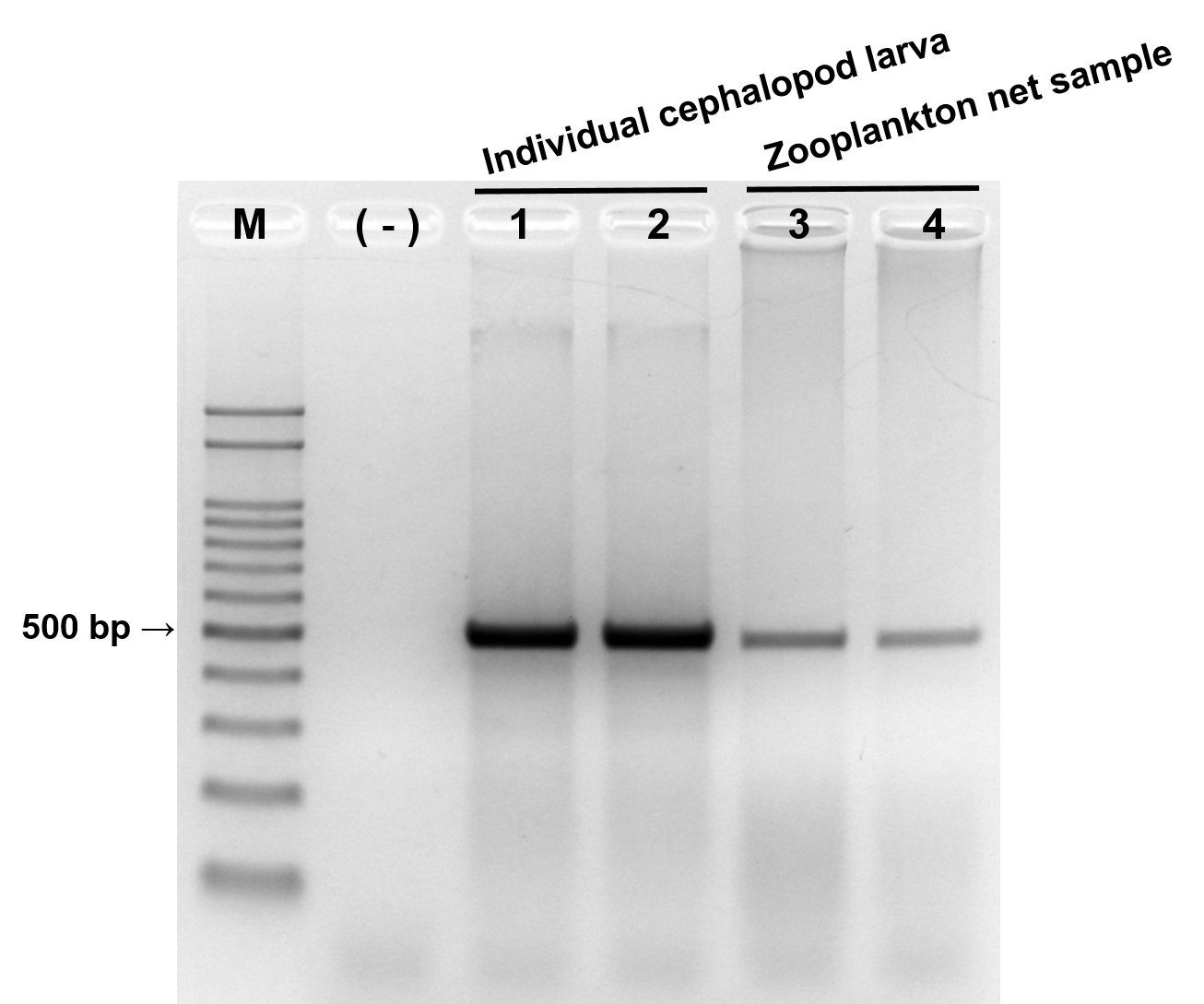

Supplement: Supplemental Information 3 — Single PCR product amplified by CPD primers using two individual cephalopod larvae and two zooplankton net samples collected from Korean waters in August, 2016. M, 100 bp DNA ladder; (-), negative control; 1, Todarodes pacificus; 2, Sepiola birostrata; 3, Stn.72; 4, Stn.206. [file peerj-07-7140-s003.png]
